# Increased heterosis in selfing populations of a perennial forb

**DOI:** 10.1093/aobpla/plv122

**Published:** 2015-10-27

**Authors:** Christopher G. Oakley, Jonathan P. Spoelhof, Douglas W. Schemske

**Affiliations:** 1 Department of Plant Biology, Michigan State University, East Lansing, MI 48824-1312, USA; 2 Department of Plant Biology and W. K. Kellogg Biological Station, Michigan State University, East Lansing, MI 48824, USA

**Keywords:** *Arabidopsis lyrata*, drift load, effective population size, genetic load, genetic rescue, mating system evolution, purging, reproductive assurance

## Abstract

Quantifying the importance of random genetic drift in natural populations is central to understanding the potential limits to natural selection. One approach is to estimate the magnitude of heterosis, the increased fitness of progeny derived from crosses between populations relative to crosses within populations caused by the heterozygous masking of deleterious recessive or nearly recessive alleles that have been fixed by drift within populations. Self-fertilization is expected to reduce the effective population size by half relative to outcrossing, and population bottlenecks may be common during the transition to selfing. Therefore, chance fixation of deleterious alleles due to drift in selfing populations should increase heterosis between populations. Increased homozygosity due to fixation or loss of alleles should also decrease inbreeding depression within populations. Most populations of the perennial herb *Arabidopsis lyrata* ssp. *lyrata* are self-incompatible (SI), but several have evolved self-compatibility and are highly selfing. We quantified heterosis and inbreeding depression in two predominantly self-compatible (SC) and seven SI populations in a field common garden experiment within the species' native range and examined the correlation between these metrics to gauge the similarity in their genetic basis. We measured proportion germination in the lab, and survival and fecundity (flower and seed production) for 2 years in the field, and calculated estimates of cumulative fitness. We found 7.2-fold greater heterosis in SC compared with SI populations, despite substantial heterosis in SI populations (56 %). Inbreeding depression was >61 %, and not significantly different between SC and SI populations. There was no correlation between population estimates of heterosis and inbreeding depression, suggesting that they have somewhat different genetic bases. Combined with other sources of information, our results suggest a history of bottlenecks in all of these populations. The bottlenecks in SC populations may have been severe, but their strong inbreeding depression remains enigmatic.

## Abstract (precis)

Quantifying the effects of population bottlenecks and inbreeding on genetic variation underlying fitness in natural populations is central to understanding the potential limits to natural selection. One approach is to estimate heterosis in crosses between populations, thus revealing deleterious mutations that have become fixed within populations by random genetic drift. We estimated heterosis in selfing and outcrossing populations of *Arabidopsis lyrata*. We found massive heterosis in selfing populations, but strong heterosis even in outcrossing populations. Combined with other sources of information, our results suggest a common history of population bottlenecks, with possibly severe bottlenecks associated with the transition to selfing.

## Introduction

Understanding the causes and consequences of differentiation among natural populations is a central goal in evolutionary biology. While much attention has been given to the effects of natural selection, random genetic drift can also be important in shaping patterns of genetic variation and determining the fate of new mutations. One empirical approach to investigating the consequences of genetic drift for genetic variation related to fitness is to estimate heterosis in crosses between populations (Crow 1948; Fenster 1991; Lynch 1991; Whitlock *et al*. 2000; Oakley and Winn 2012; Oakley *et al*. 2015). An increase in progeny fitness following between-population relative to within-population crosses is thought to be caused by the heterozygous masking (in the F_1_) of mildly deleterious, partly recessive alleles historically fixed by drift in the parental populations. While overdominance at individual loci is another possible genetic mechanism underlying heterosis, it is generally thought to be uncommon (Charlesworth and Willis 2009). Increased performance or yield in crosses due to heterosis has long been of interest in plant and animal breeding (Hochholdinger and Hoecker 2007; Lippman and Zamir 2007; Chen 2010), but its value for studying the causes and consequences of reductions in effective population sizes in natural populations has been underappreciated.

One scenario where patterns of heterosis might be informative is the transition from outcrossing to self-fertilization (Busch 2006; Willi 2013), which has fascinated evolutionary biologists since Darwin (1876, 1877). Despite over a century of interest, there are many persistent questions about the initial forces leading to shifts towards self-fertilization (Busch and Delph 2012; Wright *et al*. 2013; Arunkumar *et al*. 2015), and the subsequent evolutionary dynamics of mating system evolution (Goodwillie *et al*. 2005; Oakley *et al*. 2007; Winn *et al*. 2011).

One predicted consequence of selfing is an up to 50 % (with complete selfing) reduction in effective population size owing to reduced recombination and increased linkage disequilibrium (Pollak 1987; Nordborg 2000). Because the efficacy of selection is a function of the effective population size (Kimura 1962; Crow and Kimura 2009), a transition to selfing could result in greater fixation of deleterious mutations. Theory suggests that in very small populations, fixation of deleterious mutations can contribute to declining population sizes and eventual extinction (Heller and Smith 1978; Lynch *et al*. 1995). Chance fixation of deleterious mutations and the stochastic loss of adaptive genetic variation needed to respond to fluctuating or changing environments (e.g. Gomulkiewicz and Houle 2009) are two complementary explanations for why self-fertilization may be an evolutionary dead end (Dobzhansky 1950; Stebbins 1957; Takebayashi and Morrell 2001; Goldberg *et al*. 2010; Glémin and Ronfort 2013; Igić and Busch 2013; de Vos *et al*. 2014; Lande and Porcher 2015).

In addition to the effects of selfing *per se*, population bottlenecks associated with the transition to self-fertilization can have lasting consequences for effective population size. Selfing is thought to provide a short-term advantage during colonization (or more generally whenever outcross pollen is limited) because it provides reproductive assurance (Baker 1967; Busch and Delph 2012; Cheptou 2012), but random genetic drift associated with population bottlenecks will also lead to increased homozygosity and fixation of deleterious alleles. There is recent empirical interest in whether reductions in molecular genetic variation in selfing compared with outcrossing populations can be explained by mating system alone (because of an up to 50 % reduction in effective population size due to selfing), or if selection for reproductive assurance via population bottlenecks need be invoked (reviewed in Busch and Delph 2012; Wright *et al*. 2013).

Quantifying heterosis in crosses between both selfing and outcrossing populations may provide additional insight. Differences in heterosis between selfing and outcrossing populations can corroborate differences in molecular genetic variation and, if dramatically >2-fold in selfing compared with outcrossing populations, could suggest additional factors (like bottlenecks) beyond the effects of mating system *per se*. Additionally, absolute values of heterosis in outcrossing populations are a valuable benchmark for evaluating differences between selfing and outcrossing populations. For example, if both types of populations have experienced bottlenecks, a >50% reduction in molecular genetic variation in selfing compared with outcrossing populations might be more difficult to detect.

Both mating system and population demography can contribute to genetic differentiation between populations, but they will also affect patterns of inbreeding depression within populations. Inbreeding depression, the reduction in fitness of inbred relative to outbred progeny caused by the increased homozygous expression of partly recessive deleterious alleles, plays a central role in mating system evolution (Lloyd 1979; Lande and Schemske 1985; Schemske and Lande 1985; Charlesworth and Charlesworth 1987; Husband and Schemske 1996; Goodwillie *et al*. 2005; Charlesworth and Willis 2009; Winn *et al*. 2011). Inbreeding depression is commonly invoked to counter the evolution of self-fertilization, but may be purged with repeated selfing because of the increased efficacy of selection against nearly recessive alleles in the homozygous state (Lande and Schemske 1985), assuming a very large population. The ability of a population to selectively purge deleterious genetic variation, and in particular mildly deleterious variation, will decrease with decreasing effective population size (Kimura *et al*. 1963; Bataillon and Kirkpatrick 2000; Whitlock 2002; Glémin 2003).

Although heterosis and inbreeding depression are both thought to be caused by recessive or partly recessive deleterious alleles (e.g. Charlesworth and Willis 2009), empirical comparisons of their genetic bases are scant. A first step could be to examine the correlation between population level estimates of heterosis and inbreeding depression, though few studies estimate both metrics in the same environment for many populations (but see Paland and Schmid 2003; Oakley and Winn 2012; Willi 2013). There are theoretical expectations that they will have somewhat different genetic bases, and that population bottlenecks and self-fertilization will affect these metrics differently. Inbreeding depression is due to segregating variation within a population for both highly recessive lethal and semi-lethal alleles that segregate at low frequency, as well as more mildly deleterious, partly recessive alleles (Simmons and Crow 1977; Charlesworth and Willis 2009). Heterosis, in contrast, is caused by the heterozygous masking of mildly deleterious, partly recessive alleles that have become fixed within populations (Whitlock *et al*. 2000). Despite the expectation that many slightly deleterious alleles are likely to be stochastically lost during a bottleneck because of their low initial frequencies, an appreciable number are expected to drift to fixation. One critical distinction between heterosis and inbreeding depression then is that while at least some component of inbreeding depression may be selectively purged (Lande and Schemske 1985), or lost (Glémin 2003) and/or fixed (Whitlock 2002) by drift during a bottleneck, the deleterious alleles that cause heterosis cannot easily be purged.

Populations of *Arabidopsis lyrata* ssp. *lyrata* (Brassicaceae) in North America present an interesting opportunity to examine patterns of heterosis and inbreeding depression as a function of mating system. Most populations are predominantly self-incompatible (SI), though most contain some partially self-compatible (SC) individuals (Mable *et al*. 2005; Mable and Adam 2007; Foxe *et al*. 2010; Willi and Määttänen 2010). Self-incompatibility is thought to be ancestral, and the closely related *A. lyrata* ssp. *petraea* native to Europe is also SI. In the Great Lakes region of North America, however, moderate to high population mean self-compatibility (hereafter referred to simply as SC) has been documented for several populations and is thought to have evolved independently multiple times (Hoebe *et al*. 2009; Foxe *et al*. 2010; Willi and Määttänen 2010). Population proportion SI has been shown to be strongly positively correlated with multilocus outcrossing rates (Foxe *et al*. 2010), meaning that transitions to SC generally result in increased selfing. Populations that are highly selfing do not exhibit floral morphologies typically associated with increased selfing, e.g. reduced flower size (Foxe *et al*. 2010), which could indicate that these transitions are relatively recent.

Previous studies using molecular genetic variation to investigate the potential role of bottlenecks in the transition to selfing for North American populations of *A. lyrata* came to opposite conclusions. Foxe *et al*. (2010) reported that reduced genetic variation within selfing populations is consistent with the 50% reduction in effective population size expected due to selfing alone (Nordborg 2000), but another study (Willi and Määttänen 2011) concluded that the reduction in genetic variation was much >50%. One potential issue in using molecular data to detect signatures of bottlenecks in SC relative to SI populations is if SI populations have also experienced bottlenecks, reducing their absolute levels of variation. The Great Lakes region occupied by *A. lyrata* was glaciated during the Pleistocene and molecular evidence suggests reduced genetic diversity in this region compared with populations in the centre of the range (Griffin and Willi 2014). There is also evidence of an older (and strong) bottleneck for *A. lyrata* ssp. *lyrata* associated with colonization of North America and divergence from *A. lyrata* ssp. *petraea* (Ross-Ibarra *et al*. 2008). A shared history of population bottlenecks between selfing and outcrossing populations might make it difficult to draw conclusions about relative differences in genetic variation due to mating system.

Willi (2013) examined heterosis and inbreeding depression in *A. lyrata* ssp. *lyrata* (from the Great Lakes region) with respect to mating system in a common garden experiment in Europe. Heterosis was ∼6-fold stronger for SC (this grouping included one population with intermediate SC) than for SI populations, suggesting that some mechanism other than the mating system *per se* was responsible for a reduction in effective population. Additionally, she found that inbreeding depression was similar between SI and SC populations, and was surprisingly weak overall. Outcrossing SI populations would be expected to have strong inbreeding depression (Husband and Schemske 1996), as was found for European populations of *A. lyrata* ssp. *petraea* (Sletvold *et al*. 2013). It is well known that the environment can affect the expression of inbreeding depression (Dudash 1990; Armbruster and Reed 2005; Cheptou and Donohue 2011) and heterosis (Armbruster *et al*. 1997; Fenster and Galloway 2000). Because Willi's (2013) study was conducted far outside of the native range of the North American populations (Switzerland), the estimates of heterosis and inbreeding depression she reports may be different from those where plants were grown in their native climate. For example, mortality was very low in her experiment because seedlings were started in the spring and were watered and shaded during the first summer. Differential fitness due to overwinter survival and summer drought under native climatic conditions could influence estimates of heterosis and inbreeding depression.

Here, we report estimates of heterosis and inbreeding depression for Great Lakes *A. lyrata* grown in a 2-year field experiment located within their native range in Michigan, USA. These experiments parallel those of Willi (2013) in many respects and, therefore, offer a unique opportunity to assess the generality of the results of our studies. Like Willi (2013), we estimated heterosis and inbreeding depression for both SC and SI populations, and present data for populations in both categories, some of which are unique to our study (Table 1; cf. Willi 2013). We address the following questions: (i) What are the magnitudes of heterosis in SC and SI populations? (ii) How does the magnitude of heterosis vary across the life cycle, and what might that tell us about its genetic basis? (iii) What is the magnitude of cumulative inbreeding depression in SC vs. SI populations, and what is the relationship between population mean inbreeding depression and heterosis?

**Table 1.** Names and locations of populations used in the study, population mean (SE) estimates of proportion SI and mating system categories (SC, predominantly self-compatible; SI, predominantly self-incompatible), *n* = 6 lines for each population with 20 self-pollinations per line. Previously published estimates of outcrossing rates for these populations are also given (or n/s if no estimates are available). If multiple published estimates were available, we chose the estimate with the smallest standard error. ^1^Also included in Willi's (2013) study on inbreeding depression and heterosis. ^2^Mable and Adam (2007). ^3^Willi and Määttänen (2010).

Population^1^	Latitude	Longitude	Proportion SI (SE)	Mating system category	Outcrossing rate (SE) and reference
Rondeau, Ontario^1^	42°16′	81°51′	0.08 (0.05)	SC	0.29 (0.11)^2^
Point Pelee, Ontario	41°56′	82°31′	0.17 (0.13)	SC	0.02 (0.03)^2^
Pinery, Ontario	43°16′	81°50′	0.65 (0.11)	SI	0.84 (0.12)^2^
Bruce Peninsula, Ontario	43°12′	81°35′	0.79 (0.14)	SI	0.89 (0.08)^2^
Ludington, Michigan^1^	44°01′	86°29′	0.82 (0.10)	SI	1.03 (0.06)^3^
Indiana Dunes, Indiana^1^	41°37′	87°13′	0.88 (0.11)	SI	0.91 (0.04)^3^
Hoffmaster, Michigan	43°07′	86°16′	0.89 (0.03)	SI	n/a
Saugatuck, Michigan^1^	42°42′	86°12′	0.92 (0.05)	SI	0.97 (0.02)^3^
Rosy Mound, Michigan	43°01′	86°13′	0.98 (0.02)	SI	n/a

## Methods

### Study species and source populations

*Arabidopsis lyrata* ssp. *lyrata* (Brassicaceae) is a short-lived perennial native to North America, where it often grows in sandy or rocky soils (Mitchell-Olds 2001). Seeds typically germinate in the fall, and plants overwinter as a rosette, followed by spring flowering (Remington *et al*. 2013). We obtained *A. lyrata* seed from nine populations in the Great Lakes region, including two populations previously reported as SC and five populations reported as SI (Table 1; Foxe *et al*. 2010; Willi and Määttänen 2010). The mating system for the two remaining populations (Table 1; Hoffmaster State Park, and Rosy Mound Natural Area, Michigan) is unknown. Each of these nine populations was visited between 2006 and 2011. For each population, seeds were collected from at least nine maternal lines separated by at least 3–10 m (depending on population size).

To get plants for crossing, seeds from 8 to 12 maternal lines per population were sterilized (September 2011) in a solution of 30 % household bleach and 1 µL mL^−1^ Triton X-100 for 10 min, and sown on agar containing Gamborg's B-5 medium. Seeds were stratified for 1 week in the dark at 4 °C to break seed dormancy and synchronize germination of viable seeds. After 17 days, seedlings were transplanted from agar plates into SC10 (3.8 × 21 cm, 164 mL volume) Cone-Tainers™ (Stuewe & Sons, Inc., Tangent, OR, USA) filled with peat-based potting medium and moved into a greenhouse heated to a minimum temperature of 20 °C (high temperatures varied depending on the weather) with supplemental sodium-vapour lighting providing 16 h of daylight. The plants were sub-irrigated as needed with water containing 100 p.p.m. nitrogen fertilizer (18-9-18, pH reducing formula).

### Estimating the degree of self-compatibility

As some of the populations have no published information on degree of self-incompatibility, we quantified degree of SI for all populations in this study under the same conditions. We performed a set of 20 self-pollinations on each of 5 lines, for each of the 9 populations. We first raised plants derived from bulked outcrossed seed from the same maternal lines used in the heterosis assay (described below). We then performed self-pollinations at anthesis by applying pollen (from other flowers on the same plant) with fishing line until the stigma was visibly covered. In sporophytic self-incompatibility, pollen with SI alleles matching those of the maternal plant are prevented from growing down the style (Nasrallah and Nasrallah 1993); thus, we estimated the proportion SI for each line as one minus the proportion of flowers that set fruits (i.e. produced at least one seed; mean seed number per fruit was ∼18). This approach has been used previously to determine population SI/SC categories in this species (Mable *et al*. 2005; Foxe *et al*. 2010; Willi and Määttänen 2010).

### Crosses: heterosis

To generate seed for the field experiment to estimate heterosis, controlled hand pollinations were performed in spring of 2012. Both within- and between-population crosses were performed. We emasculated flowers just before anthesis and pollinated them with pooled pollen from four haphazardly selected lines within the same population (within-population crosses) and from four different populations (between-population crosses), respectively. Using four pollen donors for each cross for a given day ensured that the fitness effects of crosses on a given maternal plant were averaged over many different paternal plants, ensuring that the effects of between-population crosses were not specific to a particular population pair. This was also done to minimize the chance for SI effects in within-population outcrosses. To achieve this, separate pools of pollen for each line and cross type were collected and mixed in microcentrifuge tubes each morning, and transferred to stigmas as described above. An average of about nine pollinations were performed for each maternal line for each cross type (range = 4–12). Control emasculations were performed on one plant per population per day (234 in total), and none of the control emasculations set seed.

### Crosses: inbreeding depression

Controlled pollinations for estimating inbreeding depression were performed in a similar fashion, with the exception that all pollinations were done at the bud stage (Kearns and Inouye 1993). Because the expression of self-incompatibility factors begins just shortly before anthesis (Nasrallah and Nasrallah 1993), doing crosses at the bud stage allowed us to bypass the SI mechanism. Because of limited numbers of available flowers, these pollinations were done after those for estimating heterosis, and were performed on a unique set of three lines per population. Outcross pollinations were done using pooled pollen as described above. Self-pollinations were performed using pollen from other flowers on the same maternal plant. An average of 7.2 pollinations (range = 2–21) were performed for each maternal line by cross type combination.

All fruits were harvested just prior to dehiscence, and in August 2012, an average of 39 (range = 22–40) seeds per maternal line–cross type combination for the heterosis experiment, and an average of 19 (range = 7–20) seeds per combination for the inbreeding depression experiment, were sterilized and grown on agar as described above. Proportion of seeds that germinated was scored for all maternal line–cross type combinations. The seedlings were then transplanted into 6.4-cm square pots containing peat-based potting medium and grown in a growth chamber under 16 h days at 22 °C for 21 days before being transported to the site of the field experiment (42°16′N; 85°23′W—Kellogg Biological Station, Hickory Corners, MI, USA).

### Field common garden experiment

The plants were allowed to acclimate outdoors for 10 days, after which plants were transplanted into three replicate blocks in a stratified (by block) random design. The planting beds were covered with landscape fabric with holes for the plants every 31 cm. In each block, we planted an average of 7.0 (range = 6–7) and 2.9 (range = 2–3) plants per line per cross type for the heterosis and inbreeding depression experiments, respectively, for a total of 1950 seedlings. Plants were watered once shortly after transplanting, and once again in July 2013 during a prolonged dry period. Fertilizer was not used in the field experiment, and no attempt was made shade the plants, or to control pests. To facilitate censusing, we occasionally removed weeds by clipping around the base of the study plants. While the field common garden was not representative of the native habitat in terms of soil type, plants were exposed to climatic conditions (summer drought and winter freezing) typical of the region (cf. Willi 2013).

We scored three fitness components in the field: survival to reproduction (i.e. survival up to anthesis of the first flower experiment-wide), flower number and seed number per fruit in each of 2 years (2013 and 2014). Plants produced their first flowers between mid-April and mid-May, with flower production typically slowing by mid-June. All reproductive stems were harvested and bagged at the beginning of July after most new flower production had ceased. In the first year, we counted total number of flowers produced for every plant as the number of pedicels. In the second year, many of the remaining plants were quite large, so with the exception of smaller plants (see below), we estimated total pedicel number by counting the total number of pedicels on four inflorescences (selected by holding the plant upside down, and haphazardly picking four stems, without respect to inflorescence size) and multiplying mean pedicel number per inflorescence by the total number of inflorescences. For small plants (<5 inflorescences or <100 pedicels), we counted total pedicel number for the whole plant. To estimate mean seed number per fruit, we counted the total seed number in two representative (average sized) siliques per plant for three plants per cross type per line and block in the first year. Many fewer plants survived to reproduce in the second year, so we sampled fruits from only one plant per line–type combination per block in the second year.

### Calculating cumulative fitness

Cumulative fitness (seeds per seed sown) after 1 year was calculated for each maternal line–cross type combination as the product of proportion germination, proportion survival to reproduction in 2013, total number of pedicles in 2013 and average seed number per fruit in 2013. Based on a subset of plants (*n* = 955, approximately balanced across populations and cross types) in 2013, there was a strong and significant correlation between line–cross type mean number of pedicels and line–cross type mean total number of fruits produced for both SC (*r* = 0.99, *P* < 0.001) and SI (*r* = 0.95, *P* < 0.001) populations, so the use of pedicle number in calculating cumulative fitness is not unreasonable. Cumulative fitness over 2 years was calculated as fitness in Year 1 plus the product of proportion germination, proportion survival to reproduction in 2013, proportion survival to reproduction in 2014, total number of pedicles in 2014 and average seed number per fruit in 2014. Over 99 % of plants that survived to the experiment-wide start of flowering in a given year flowered, so we omit probability of reproduction as a fitness component. We also omitted seed number per fruit (from the initial crosses) from our estimate of cumulative inbreeding depression because greater failure of *in situ* pollen tube growth in bud selfs compared with bud outcrosses for SI populations (J. P. Spoelhof, unpubl. data) indicates that we could not completely distinguish early inbreeding depression from self-incompatibility.

### Statistical analyses

The effect of population on proportion SC was analysed with a one-way analysis of variance (ANOVA) using line mean proportion SC. For the heterosis comparison, fitness components and cumulative fitness were analysed using mixed-model ANOVA. Mating system category (SC vs. SI), cross type (within population vs. between population) and their interaction were treated as fixed effects. Population nested within mating system category, the interaction between this term and cross type, and line nested within population and mating system category were all treated as random effects. Significance of random effects was tested using likelihood ratio tests comparing models with and without the term of interest, which are approximately χ^2^ distributed with one degree of freedom. All analyses were performed using line means for each cross type. All statistical analyses were performed using JMP v. 11 (JMP 2013). For fitness components measured in the field, we first calculated block means of each line–cross type combination, and then calculated line–cross type means as the mean of the block means. This allowed us to account for spatial variation among blocks, and to treat survival as a continuous variable (the full ANOVA model treating survival as a binary response using individual data failed to converge). Limited sample sizes within each block for each line–cross type combination for scoring survival as a binary trait also precluded the use of more sophisticated analyses of cumulative fitness (e.g. Aster models; Shaw *et al*. 2008).

Because we are interested in differences in ratios (heterosis) between mating system categories, it is appropriate to use natural-log-transformed data (Johnston and Schoen 1994). We report results on untransformed data because of generally better model fit and ease of interpretation of the resultant least square means (LSMs). However, re-analysis with log-transformed (after adding a small constant of one fruit to deal with plants with zero fitness) data gave qualitatively similar results for the cross type and the interaction between mating system and cross type terms (results not shown) with one exception—a significant interaction between cross type and mating system category was detected for the log-transformed data and not the untransformed data for survival in 2014.

For inbreeding depression, we had lower sample sizes overall, particularly for components of fecundity in the second year, so we focussed on differences among mating system-cross type combinations for cumulative fitness. We conducted an analysis on cumulative fitness over 2 years as described above, using two cross types (bud selfs vs. bud outcrosses within populations). These data were analysed separately from the heterosis experiment because unique maternal and paternal lines were used for the pollinations, and because of differences in the pollination technique (bud pollinations were necessary for the inbreeding depression experiment).

Heterosis was calculated as [(*W*_between_ − *W*_within_)/*W*_within_× 100] (Whitlock *et al*. 2000). Heterosis has sometimes previously been calculated using the fitness of between-population crosses in the denominator (e.g. van Treuren *et al*. 1993; Ouborg and Van Treuren 1994; Oakley and Winn 2012). This implies that outcrossing between populations is the ‘optimal’ fitness, and that there is a ‘load’, or fitness cost, of crossing within populations. This ‘load’ is unrealistic, particularly for populations that are naturally spatially structured such that gene flow between populations is low. Using fitness of within-population outcrosses in the denominator, as we do here, is more straightforward. Heterosis quantifies the relative strength of drift among natural populations using mean fitness within populations as the frame of reference. Inbreeding depression was calculated as [(*W*_bud outcross_ − *W*_bud self_)/*W*_bud outcross_× 100] (Lande and Schemske 1985). Inbreeding depression is the parameter most relevant to contemporary mating system evolution, so we present it rather than inbreeding load (standardizing based on population inbreeding coefficients) as was done by Willi (2013). The relationship between population level estimates of heterosis and inbreeding depression for cumulative fitness was examined using Spearman's rank correlation because of the very different distributions of values of heterosis and inbreeding depression.

## Results

### Population level estimation of proportion self-compatibility

As expected, there was significant variation among populations in proportion SI (*F*_8,36_ = 13.2, *P* < 0.001, *R*^2^ = 0.75). Seven populations had average proportions SI from 0.65 to 0.98, and were thus classified as SI, while the two remaining populations had SI estimates of 0.08 and 0.17, and were classified as SC (Table 1). Previously published outcrossing rate estimates for five of the seven SI populations averaged 0.93 (range = 0.84–1.03), and were 0.29 and 0.02 for the two SC populations (Table 1).

### Heterosis: germination

**Figure 1.**
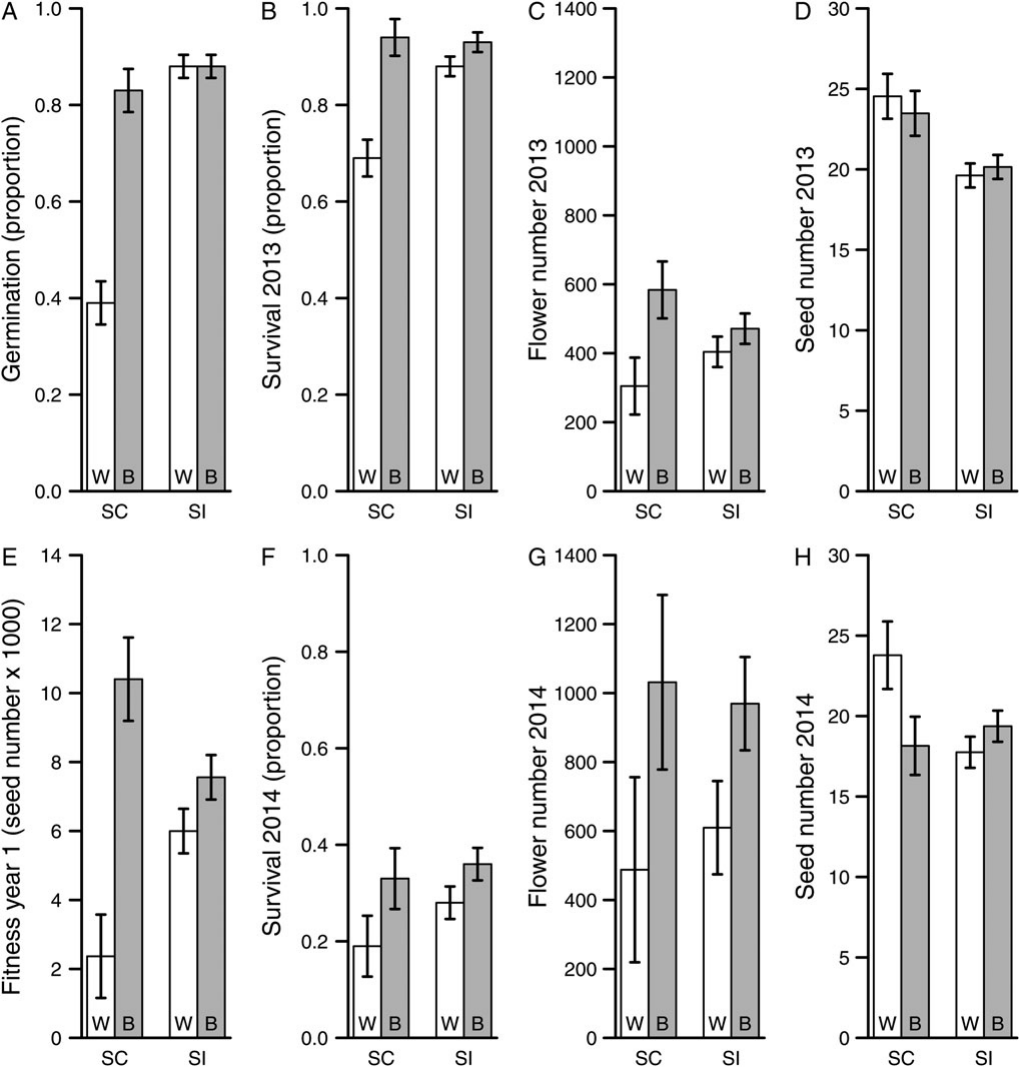
Least square mean values (±1 SE) for the heterosis experiment. Fitness components (A–D; F–H) and cumulative fitness in the first year (E) are given for each mating system category (SC, self-compatible; SI, self-incompatible) and cross type (W, within-population crosses; B, between-population crosses).

Overall, proportion germination of the 2833 seeds sown was 0.82 (range = 0.18–1.00). Mating system category, cross type and their interaction all had highly significant effects on proportion germination, as did the effect of line nested within population and mating system category (Table 2). Mean proportion germination was virtually identical between cross types for SI (−0.4 % heterosis; Fig. 1A), but for SC, between-population crosses had significantly greater germination (*P* < 0.001 for comparison of LSMs) than within-population crosses (111 % heterosis; Fig. 1A).

**Table 2.** Heterosis: ANOVA for proportion germination (Germ.) measured in the lab, proportion survival, total flower production and seed number per fruit measured in the field (2 years) and cumulative fitness (total seeds produced per initial seed sown). Table entries for fixed effects are *F* values (df = 1,7), and table entries for random effects are χ^2^ values. *P < 0.05, **P < 0.01, ***P < 0.001.

ANOVA effect	Germ.	Year 1			Year 2			Cumul. fitness (Year 1)	Cumul. fitness (Years 1 and 2)
		Survival	Flowers	Seeds/fruit	Survival	Flowers	Seeds/fruit		
Fixed effects									
SC category	36.68***	7.90*	0.01	8.79*	0.77	0.01	2.33	0.12	0.79
Cross type	71.87***	25.67**	22.68**	0.12	17.58**	9.30*	1.74	37.55***	114.86***
SC cat. × cross type	74.26***	12.52**	8.49*	1.11	2.00	0.39	5.70*	17.10**	22.81**
Random effects									
Population (SC cat.)	0.01	0.08	3.79	1.19	4.66*	2.04	0.06	0.66	3.78
Pop. × cross type (SC cat.)	0.08	0.12	3.12	0.07	0.62	0.30	0.07	3.13	0.01
Line (Pop., SC cat.)	7.97**	0.04	5.17*	0.16	0.14	0.00	0.48	0.77	0.04

### Heterosis: Year 1

Overall average maternal line–cross type proportion survival to reproduction was 0.88, but ranged from as low as 0.41 to up to 1. Cross type, mating system category and their interaction all had significant effects on proportion survival (Table 2). For SI, between-population crosses had slightly but not significantly (*P* = 0.15) greater survival than within-population crosses (5 % heterosis; Fig. 1B). For SC in contrast, between-population crosses had significantly higher survival (*P* = 0.002) than within-population crosses (37 % heterosis; Fig. 1B).

Of the survivors, average number of flowers produced in 2013 was 439 (range = 167–935), and the average number of seeds per fruit was 20.8 (range = 11.7–28.7). Both cross type and the interaction between mating system and cross type had significant effects on flower number (Table 2), as did line nested within population and mating system category. Between-population crosses had greater average flower production than within-population outcrosses for both SI and SC (17 and 92 %, heterosis respectively; Fig. 1C); these differences were significant for SC (*P* = 0.003), but only suggestive for SI (non-significant *P* = 0.09). For average seed number per fruit, the only significant effect was mating system category (Table 2), SC producing ∼21 % more seeds per fruit than SI (Fig. 1D).

Cumulative fitness up to the end of the first year of the experiment (seeds per seed sown) for the various line and cross type combinations averaged 2784 seeds overall but was extremely variable, spanning three orders of magnitude (range = 6–8629). Both cross type and the interaction between mating system category and cross type had significant effects on mean fitness (Table 2). For SC, between-population outcrosses had significantly (*P* < 0.001) and dramatically greater fitness than within-population outcrosses (339 % heterosis; Fig. 1E). In contrast, for SI, there was only a slight (26 % heterosis; Fig. 1E), non-significant (*P* = 0.07) advantage to between-population outcrosses.

### Heterosis: Year 2

Average maternal line–cross type proportion survival to reproduction in 2014 (of the 1316 remaining plants) was 0.31 (range = 0.00–0.61). There was a significant main effect of cross type on proportion survival (Table 2), between-population outcrosses had 47 % higher proportion survival than crosses within populations (Fig. 1F). While there was no significant mating system by cross type interaction, on average, SC had greater heterosis for survival than did SI (79 vs. 26 %). Of the 408 surviving plants, average overall number of flowers produced was 791 with a >50-fold range (39–2247). The only significant effect on flower production was cross type (Table 2), between-population outcrosses produced ∼82 % more flowers than within-population crosses (Fig. 1G). It should be noted that there was limited statistical power for fecundity components in 2014 because of the relatively small number of plants surviving from Year 1 to Year 2. Although there was no significant mating system by cross type interaction, on average, SC had greater heterosis for flower production than did SI (111 vs. 59 %). For average seed number per fruit, the only significant effect was the interaction between mating system category and cross type (Table 2). Within- and between-population crosses produced similar numbers of seeds per fruit for SI (9 % heterosis; Fig. 1H), but for SC, between-population crosses had 24 % fewer (outbreeding depression) seeds per fruit than within-population crosses.

### Heterosis: cumulative fitness

**Figure 2.**
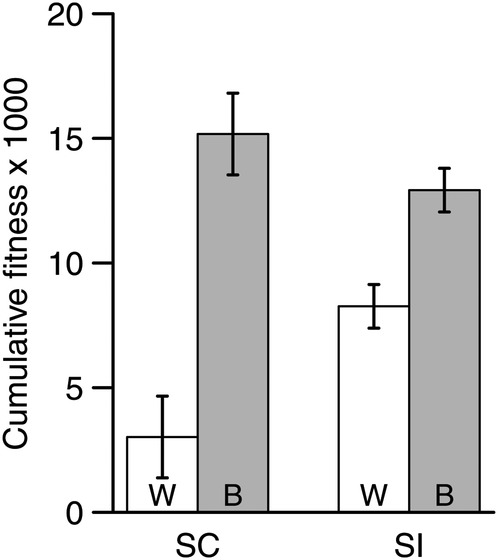
Least square mean (±1 SE) cumulative fitness over 2 years for the heterosis experiment. Least square means are given by mating system category (SC, self-compatible; SI, self-incompatible) and cross type (W, within-population crosses; B, between-population crosses).

For cumulative fitness over all stages for the entire 2 years of the experiment, overall average maternal line–cross type number of seeds per seed sown was 10 261, with ∼60-fold variation in the range of maternal line–cross type mean estimates (355–21 580). The effects of cross type and its interaction with mating system category were highly significant (Table 2). Between-population crosses had higher cumulative fitness than within-population crosses in both SC and SI (*P* < 0.001 for both LSM comparisons), but the magnitude of heterosis was much greater in SC than in SI (402 vs. 56 %; Fig. 2). Heterosis was 7.2 times greater in SC compared with SI populations, and the lower confidence interval on the difference in heterosis between SC and SI populations was 398 % of the mean heterosis for the SI group, suggesting that at minimum, heterosis is nearly 4-fold greater in SC than in SI populations.

### Inbreeding depression

**Figure 3.**
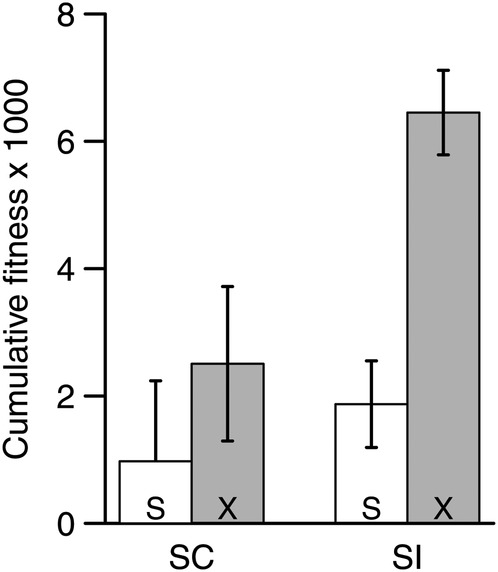
Least square mean (±1 SE) cumulative fitness over 2 years for the inbreeding depression experiment. Least square means are given by mating system category (SC, self-compatible; SI, self-incompatible) and cross type (S, bud selfed; X, bud outcrossed).

For the inbreeding depression experiment, overall cumulative fitness was 3678 seeds per seed sown with a range spanning almost three orders of magnitude (12–11 205). Cross type was the only model term with a significant effect on fitness (Table 3). Overall, outcrosses had ∼68 % greater fitness than progeny derived from selfing (Fig. 3). Both SI and SC had similar levels of inbreeding depression (71 and 61 %, respectively), and there was no significant interaction between mating system category and cross type (Table 4). Population level estimates of inbreeding depression and heterosis were not significantly correlated (*r*_s_ = 012, *P* = 0.77; Table 4).

**Table 3.** Inbreeding depression: ANOVA for cumulative fitness (total seeds produced per initial seed sown over the entire experiment). Table entries for fixed effects are *F* values, and table entries for random effects are χ^2^ values. **P = 0.008.

ANOVA effect	Cumulative fitness
Fixed effects	
SC category	4.48
Cross type	13.78**
SC cat. × cross type	3.43
Random effects	
Population (SC cat.)	0.91
Pop. × cross type (SC cat.)	0.86
Line (Pop., SC cat.)	0.28

**Table 4.** Population level heterosis and inbreeding depression for cumulative fitness estimates. Values are given as percentages for each population in each mating system category (SC, predominantly self-compatible; SI, predominantly self-incompatible).

Population	Cumulative year 1		Cumulative total	
	Heterosis	Inbreeding depression	Heterosis	Inbreeding depression
SC				
Rondeau	221	71	292	65
Point Pelee	519	40	484	67
SI				
Pinery	41	77	85	88
Bruce Peninsula	28	78	61	72
Luddington	82	63	93	75
Indiana Dunes	5	50	43	58
Hoffmaster	22	68	45	73
Saugatuck	8	67	40	68
Rosy Mound	36	61	63	64

**Figure 4.**
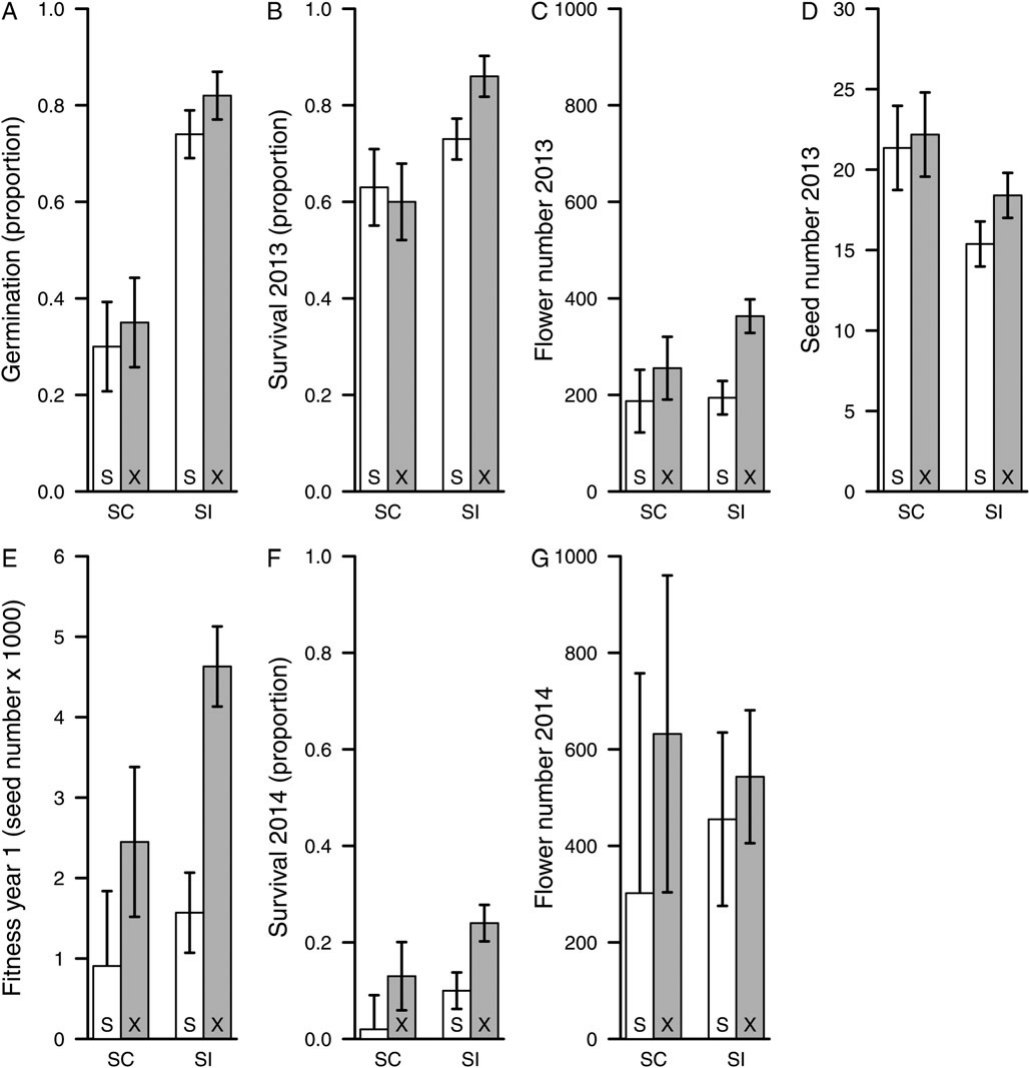
Least square mean values (±1 SE) for fitness components and cumulative fitness in the first year for the inbreeding depression experiment. Least square means are given by mating system category (SC, self-compatible; SI, self-incompatible) and cross type (S, bud selfed; X, bud outcrossed).

For stagewise estimates of inbreeding depression and cumulative fitness after the first year, there were no significant mating system category by cross type interactions (Table 5). As with cumulative fitness, inbreeding depression was similar in SC and SI populations for fitness components (Fig. 4). Significant effects of cross type for flowers in Year 1, survival to Year 2 and cumulative fitness in Year 1 (Table 5) indicate overall inbreeding depression for these components (Fig. 4C, E and F).

**Table 5.** Inbreeding depression: ANOVA for fitness components (proportion germination in the lab, and proportion survival, total flower production and seed number per fruit in the field for 2 years) and cumulative fitness in the first year. Table entries for fixed effects are *F* values, and table entries for random effects are χ^2^ values. Model terms that could not be estimated are indicated with n/a. *P < 0.05, **P < 0.01, ***P < 0.001, ^†^non-significant 0.05 < *P* < 0.10.

ANOVA effect	Germ.	Year 1			Year 2			Cumul. fitness (Year 1)
		Survival	Flowers	Seeds/fruit	Survival	Flowers	Seeds/fruit	
Fixed effects								
SC category	37.15***	5.55^†^	0.73	3.53	1.92	0.01	n/a	3.21
Cross type	0.74	1.04	15.28**	1.80	10.42*	0.50	0.00	10.93*
SC cat. × cross type	0.03	2.42	2.76	0.58	0.17	0.17	n/a	1.19
Random effects								
Population (SC cat.)	0.02	0.15	4.64*	2.77^†^	3.06^†^	2.12	0.03	0.17
Pop. × cross type (SC cat.)	0.59	0.00	0.00	0.03	0.13	n/a	0.72	1.47
Line (Pop., SC cat.)	0.17	1.29	1.78	0.90	0.67	0.06	0.05	0.12

## Discussion

### Heterosis for cumulative fitness: self-compatible vs. self-incompatible

We found dramatically greater (7.2-fold) heterosis in cumulative fitness for SC compared with SI populations despite considerable (56 %) heterosis on average for SI populations. Our average difference in heterosis between SC and SI populations is very similar to the 6.1-fold difference (289 % for SC compared with 47 % for SI) reported by Willi (2013). This is remarkable given the different methodologies and environmental conditions of the two studies—high mortality due to overwinter survival and summer drought, but high fecundity in our study vs. virtually no mortality, but more natural levels of fecundity in Willi's study (2013)—suggesting that much greater heterosis in SC populations is a general feature of populations in this region. One important limitation of our study was that we only had two SC populations, but the concordance of our results for heterosis with respect to mating system with those of Willi (2013) suggests that our conclusions are robust. Theory predicts a 50% reduction in effective population size with selfing compared with outcrossing, all else being equal (Pollak 1987; Nordborg 2000). Quantitative predictions about the amount of subsequent heterosis would depend on the distribution of selection and dominance coefficients of deleterious mutations, but the sheer magnitude of the difference observed between SC and SI populations suggests an additional mechanism beyond the effects of mating system *per se*.

Willi (2013) proposed that selfing populations are experiencing a mutational meltdown (Lynch *et al*. 1995). Under such a scenario, SC populations would be very small and caught in a negative feedback loop between the accumulation of deleterious mutations and shrinking population size. However, given that estimated census population sizes for most of the SC populations are in the range of thousands to tens of thousands (Willi and Määttänen 2011), mutational meltdown in these populations seems very unlikely (cf. Lynch *et al*. 1995). Indeed, one of the populations for which we observed strong heterosis (292 %; Rondeau, Ontario) has an estimated census size of over 1 million individuals (Willi and Määttänen 2011). One exception might be our other SC population (Point Pelee, Ontario), which was much smaller and patchier than many populations encountered, and had the strongest heterosis (484 %, Table 5).

Because all of these populations occupy regions that were glaciated during the Pleistocene and show reduced levels of neutral genetic variation compared with populations from the centre of the range (Griffin and Willi 2014), we suggest that a history of population bottlenecks is a plausible explanation for at least part of the observed heterosis. It is an intriguing possibility that (severe) bottlenecks may have played a role in the transition to greater selfing in SC populations. However, a study comparing molecular genetic diversity in SC and SI populations of *A. lyrata* ssp. *lyrata* (with many of the same populations) concluded that mating system alone was sufficient to explain relative differences in genetic diversity between SI and SC populations (Foxe *et al*. 2010). We suggest that a history of population bottlenecks in outcrossing SI populations (consistent with observed heterosis in SI populations) and/or ancestral populations of *A. lyrata* ssp. *lyrata* (Ross-Ibarra *et al*. 2008) during colonization of North America could make it difficult to detect a pattern when comparing genetic variation between SI and SC populations.

### Heterosis for cumulative fitness overall

Estimates of heterosis for overall fitness of SC populations from Willi's (2013) and the present study are, to the best of our knowledge, among the strongest reported from natural populations. Comparably strong heterosis (327 % recalculated as between-population crosses relative to within-population crosses) was observed for very small populations of an endangered plant (Oakley and Winn 2012), but heterosis is usually more modest (Fenster and Galloway 2000; Paland and Schmid 2003; Willi and Fischer 2005; Bailey and McCauley 2006; Coutellec and Caquet 2011). Perhaps the most similar study to ours reported 74 % heterosis for cumulative fitness (Busch 2006) in a small, isolated and SC population of *Leavenworthia alabamica* (Brassicaceae). This amount of heterosis is comparable with what we observe in several of our SI populations (Table 4). Unfortunately, many studies noting heterosis do not explicitly quantify it, or calculate it, in a non-standard way (see Methods). We recommend that at minimum, studies always report mean values for each of the cross types to facilitate future synthesis. We provide a text file of our LSMs by population and cross type **[see** **Supporting Information****]**.

### Heterosis for stagewise fitness components

In addition to overall fitness, examining patterns of heterosis for different fitness components can identify which fitness components are most important, and may even provide some insight into the genetic basis of heterosis. We found significant overall heterosis for all fitness components except seed number per fruit, with the greatest magnitudes observed for flower production in each year, and survival to reproduction in the second year. For SI populations, despite significant heterosis for cumulative fitness, there was no significant heterosis for any individual fitness component, highlighting the need to incorporate fitness components across the entire life cycle in estimates of heterosis. For SC populations, on average, heterosis was strongest for seed germination (111 %), and was also strong for flower number in both years, and survival to reproduction in Year 2. Very strong heterosis for seed germination in SC populations was somewhat unexpected (cf. Husband and Schemske 1996) because this trait is expressed very early in the life cycle and should thus involve fewer genes than the sum total contributing to (for example) fruit production. It is possible that more strongly deleterious mutations have become fixed at this stage, a hypothesis that could be tested in the future by linkage mapping of heterosis in crosses between SC populations. Strong heterosis for germination (110 %) has also been reported for a small and isolated SC population of *L. alabamica* (Busch 2006).

With the exception of strong heterosis for seed germination in SC populations, greater heterosis in fitness components in the second year of the experiment is consistent with the fixation of many mildly deleterious mutations that accumulate throughout the life cycle. One commonality between our results and Willi's (2013) is strong heterosis for SC populations for flower production, particularly in the second year. This is in spite of the high overall flower production in our experiment (cf. Willi 2013). Perhaps the biggest difference between the two studies is that we found modest to strong heterosis for survival to reproduction in both years, whereas Willi (2013) had very low mortality for the duration of her experiment. The (greater) heterosis we observe for survival in our study is likely due to exposure of the plants to their native climatic conditions (precipitation and winter minimum temperature). Low survival to reproduction in the second year of our experiment was unlikely a result of a trade-off with high fecundity in the previous year because mean flower number in 2013 was similar between plants that survived to reproduce in 2014 and those that did not (437 vs. 451, respectively, for SC and 404 vs. 414, respectively, for SI).

### Inbreeding depression

Inbreeding depression plays a central role in the evolution of mating systems (Lande and Schemske 1985; Goodwillie *et al*. 2005), and our estimates of inbreeding depression for cumulative fitness for both SI (71 %) and SC (61 %) populations are much stronger than previously reported for North American populations of *A. lyrata* (31 and 18 %, recalculated as inbreeding depression from Table S4; Willi 2013). Stronger inbreeding depression in our study compared with Willi's (2013) is likely attributable to differential mortality (due to winter temperatures and summer drought typical of the native range) in our study. Alternatively, stronger inbreeding depression in our study could be due to increased opportunity for selection (Waller *et al*. 2008) due to the high variance in fecundity, though it is unclear why this would affect inbreeding depression and not heterosis. Our estimates of inbreeding depression are of similar magnitude to those reported recently for two European populations of *A. lyrata* ssp. *petraea* (∼80 %; Sletvold *et al*. 2013). Inbreeding depression for fitness components was also strong in an earlier study on a single population of this species, though inbreeding depression for cumulative fitness was not reported (Kärkkäinen *et al*. 1999). In *A. lyrata* ssp. *petraea*, early inbreeding depression for seed number per fruit in the initial pollinations was particularly strong (∼60 %; Sletvold *et al*. 2013), presumably due to lethal or semi-lethal alleles, which are common in predominantly outcrossing populations and species (Husband and Schemske 1996). Because we omitted seed number per fruit from our estimates of cumulative fitness, our estimates of inbreeding depression, while strong, are likely underestimates.

Our finding of strong inbreeding depression in SC populations is unexpected. Regular selfing in large populations should purge some inbreeding depression (Lande and Schemske 1985), and published outcrossing rates for the two SC populations used here are low enough that some purging would be expected (Table 1). A recent review (Winn *et al*. 2011) found that highly selfing populations (outcrossing rate <0.2) did indeed have lower average inbreeding depression, but there was no clear pattern for more intermediate levels of selfing. Selective interference, whereby plants may produce the majority of their progeny by selfing but very few of the selfed progeny survive and reproduce, could explain higher than expected levels of inbreeding depression in predominantly selfing populations (Lande *et al*. 1994; Winn *et al*. 2011). However, previously published estimates of inbreeding coefficients (Mable and Adam 2007) for the two SC populations in our study (Rondeau = 0.52, Point Pele = 0.40) are not consistent with selective interference, where inbreeding coefficients are expected to be close to zero (Lande *et al*. 1994; Winn *et al*. 2011).

The strong inbreeding depression in SC populations is even more curious given that these populations also exhibit strong heterosis. The absence of a significant negative correlation between inbreeding depression and heterosis indicates that they have at least partially different genetic bases, but little inbreeding depression would be expected in populations where strong genetic drift had caused many segregating deleterious mutations to become lost or fixed (Pujol *et al*. 2009; Barringer *et al*. 2012). We cannot provide a conclusive explanation for our finding of both strong inbreeding depression and heterosis, but we offer a few possibilities: First, a high mutation rate of deleterious mutations after the recovery from the putative bottlenecks could result in some of the inbreeding depression observed. Second, during such bottlenecks, some (strongly) deleterious alleles may have been driven to high frequency, and now contribute to inbreeding depression. Finally, our estimates of inbreeding depression could be inflated because of unusually high variance in fecundity in our experiment, but feel that similar results between our study and Willi's (2013) for heterosis suggest that such an effect is unlikely to be strong.

## Conclusions

In summary, we find considerable heterosis in all populations, consistent with a history of population bottlenecks during post-Pleistocene range expansion. The >7-fold increase in heterosis in SC compared with SI populations may be explained by differences in mating system, and in part to strong population bottlenecks during the transition to self-fertilization. Stagewise estimates suggest that some of the heterosis may be caused by strongly deleterious alleles, and highlight the importance of estimating fitness components across the life cycle under field conditions. Estimates of heterosis for replicate selfing and outcrossing populations in other systems may provide complementary insight to patterns of molecular genetic variation for understanding the role of population bottlenecks in mating system transitions. Our estimates of cumulative inbreeding depression are much stronger than previously reported in the same study system (Willi 2013), and suggest that future work is needed to elucidate the mating system dynamics in predominantly SC populations.

## Sources of Funding

This work was funded in part by a National Science Foundation (USA) grant (Division of Environmental Biology 1022202) to D.W.S.

## Contributions by the Authors

C.G.O. and D.W.S. conceived of, and designed, the study. C.G.O. and J.P.S. conducted the study. C.G.O. analysed the data and prepared the tables and figures. C.G.O. wrote the paper with contributions from J.P.S. and input from D.W.S.

## Conflict of Interest Statement

None declared.

## Supporting Information

The following additional information is available in the online version of this article –

**LSMs_by_Population_and_CrossType.txt.** Data. Least square mean values for estimates of each fitness component and cumulative fitness separately by population and cross type.

Additional Information
