# Supplementary material for: Increased heterosis in selfing populations of a perennial forb
Source: AoB Plants. 2015 Oct 27;7:plv122. doi: 10.1093/aobpla/plv122 (PMC4671326; doi:10.1093/aobpla/plv122)
Supplement: Additional Information [file supp_7_plv122_index.html]

Increased heterosis in selfing populations of a perennial forb — Increased heterosis in selfing populations of a perennial forb — Additional Information 

# Increased heterosis in selfing populations of a perennial forb

## Additional Information

Additional Information

- Additional Information - txt file
